# Effect of cyclic, low dose pyrimethamine treatment in patients with Late Onset Tay Sachs: an open label, extended pilot study

**DOI:** 10.1186/s13023-015-0260-7

**Published:** 2015-04-17

**Authors:** Etty Osher, Aviva Fattal-Valevski, Liora Sagie, Nataly Urshanski, Nadav Sagiv, Leah Peleg, Tally Lerman-Sagie, Ari Zimran, Deborah Elstein, Ruth Navon, Avi Valevski, Naftali Stern

**Affiliations:** Institute of Endocrinology, Metabolism and Hypertension, Tel Aviv Sourasky Medical Center, Sackler Faculty of Medicine, Sackler School of Medicine, Tel Aviv University, Tel Aviv, Israel; Pediatric Neurology Unit, Sackler School of Medicine, Tel Aviv University, Tel Aviv, Israel; Tel Aviv-Sourasky Medical Center; Geha Mental Health Center, Sackler School of Medicine, Tel Aviv University, Tel Aviv, Israel; Pediatric Neurology Unit, Wolfson Medical Center, Sackler School of Medicine, Tel Aviv University, Tel Aviv, Israel; Sheba Medical Center, Sackler School of Medicine, Tel Aviv University, Tel Aviv, Israel; Department of Human Genetics, Sackler School of Medicine, Tel Aviv University, Tel Aviv, Israel; Shaare Zedek Medical Center, Hadassa School of Medicine, Hebrew University, Jerusalem, Israel

**Keywords:** Late Onset Tay Sachs, Pyrimethamine, β hexosaminidase A

## Abstract

**Background:**

Late Onset Tay- Sachs disease (LOTS) is a rare neurodegenerative lysosomal storage disease which results from mutations in the gene encoding the α subunit (HEXA) of β-hexosaminidase enzyme (HexA). At the present time, no effective treatment exists for LOTS and other neurodegenerative diseases involving the central nerve system (CNS). Pyrimethamine (PMT) was previously shown to act as a HexA chaperone in human fibroblasts *in vitro* carrying some (e.g., αG269S), but not all LOTS-related mutations. The present study assessed the effect of cyclic, low dose and long term pyrimethamine treatment on HexA in subjects with LOTS.

**Methods:**

In an open label trial in 4 LOTS patients, PMT was initiated at an average daily dose of ~2.7 mg and administered cyclically guided by blood lymphocyte HexA activity for a mean duration of 82.8 (±22.5; SD) weeks (~1.5 year).

**Results:**

HexA activity rose in all subjects, with a mean peak increase of 2.24 folds (±0.52; SD) over baseline activity (range 1.87-3). The mean treatment time required to attain this peak was of 15.7 (±4.8; SD) weeks. Following increase in activity, HexA gradually declined with the continued use of PMT, which was then stopped, resulting in the return of HexA activity to baseline. A second cycle of PMT treatment was then initiated, resulting again in an increase in HexA activity. Three of the patients experienced a measurable neuropsychiatric deterioration whereas one subject remained entirely stable.

**Conclusions:**

Cyclic low dose of PMT can increase HexA activity in LOTS patients. However, the observed increase is repeatedly transient and not associated with discernible beneficial neurological or psychiatric effects.

## Background

Late Onset Tay Sachs disease (LOTS) is a rare variant of Tay Sachs disease (TSD), that results from mutations in the gene encoding the α subunit of β-hexosaminidase A (HexA). HexA is a lysosomal enzyme which degrades the ganglioside GM2 [1], thus allowing normal GM2 turnoover. Tay-Sachs disease evolves due to accumulation of GM2 in neural cells in the central nervous system secondary to decreased HexA activity. The disease is clinically heterogeneous: in the most severe form, infantile TSD, severe Hex A malfunction leaving residual activity of just ~ <0.5% of the normal level leads to rapid neurodegeneration, culminating in infant mortality. In contrast, the LOTS variant occurs in individuals harboring compound heterozygote or mild homozygote α subunit gene mutation and is linked to higher residual HexA catalytic efficacy amounting to 2-5% of normal activity. This results in a more gradual course of neurodegeneration as evidenced by delayed onset and more gradual decline in motor, cerebral and spinocerebellar function. Additionally, LOTS is typically associated with psychiatric manifestations such as depression, bipolar disorder and psychosis [2]. Based on the correlation between residual HexA activity and the clinical phenotype in subjects carrying TSD mutations, it has been suggested that 10% of the normal HexA activity could be sufficient to avoid the development of a clinical disorder [3]. No clinically effective treatment for LOTS and other lysosomal diseases involving the central nerve system presently exists. Since any such putative mode of therapy requires access to the central nervous system (CNS), the use of small molecules functioning as pharmacological chaperones, which can potentially stabilize the native folding of the protein despite its anomalous conformation, thus preventing aggregation and restoring enzyme activity, appears attractive. Mahuran’s group pioneered the application of this approach in LOTS and tested the possibility that pharmacological chaperones can augment HexA activity in cells carrying a variety of LOTS-related mutations *in vitro* [4]. Following extensive screening of candidate molecules, these investigators identified pyrimethamine (PMT), an FDA approved anti-marial/anti-toxoplasmosis agent already in use in humans and is capable of entering the central nervous system (CNS) [5-8], as an enhancer of HexA activity *in vitro* in human fibroblasts carrying some (e.g., αG269S), but not all LOTS related mutations [9]. We have previously reported that PMT administered in a dose escalating protocol was able to enhance HexA activity in 9 patients LOTS patients who were compound heterozygotes carrying the αG269S/c.1278insTACT mutations. This may have been associated with some subtle clinical benefits. Similar biochemical effects were reported by Clarke et al. [10,11]. However, our previous results also indicated that the effect of PMT wanes and, at times, is even reversed with an increasing dose of PMT and the passage of treatment time. The present brief report, therefore, summarizes a follow up study of the biochemical effect of PMT, using a modified protocol. Here we tested the effect of cyclic low dose PMT on HexA activity in 4 out of the 9 LOTS patients included our previous study.

## Methods

### Patient recruitment and study design

Inclusion criteria were as those applied in our previous study [10], namely, genetically and clinically confirmed diagnosis of LOTS (compound heterozygotes carrying the αG269S/c.1278insTACT mutation); lack of contraindications for the use of PMT and patient’s interest in close surveillance at the study center. Exclusion criteria for participation in this study were any known sensitivity to PMT, the presence of known or convulsive disease, and any serious medical illness such as cardiac disease, hematologic abnormality, or malabsorbtion [10]. In all, 4 out of the 9 with LOTS participating in our previous study expressed interest to enroll into this modified continuation protocol and were all found eligible.

Subjects were assessed every 4–8 weeks apart by a multidisciplinary team including a neurologist, psychiatrist and a specialist in internal medicine. As part of this assessment, HexA activity was also assessed every 4–8 weeks, at each of the evaluation points. PMT was prescribed at a dose of 6.25 mg taken orally 3 times a week (~2.7 mg/day). This dose was chosen according to the results obtained from our previous study, in which PMT’s effect on HexA activity and tolerability was examined in 9 patients at a dose range reaching up to 75 mg PMT/day. In that former study, PMT at a dose of 6.25 mg taken orally 3 times a week was effective in increasing HexA activity, yet it induced no side effects [10]. In the present study we sought to evaluate the long term effect of the low dose of PMT and maintained therapy as long as no appreciable clinical or biochemical side effects were noted and HexA activity did not decline by more than 25% as compared to the pre-treatment level. Folic acid (5 mg/day) was added with the initiation of PMT. Mean duration of the active PMT treatment was 82.8 (±22.5) weeks.

The primary outcome measures were the changes in HexA activity in peripheral lymphocytes as well as the safety and tolerability of PMT therapy in LOTS patients in a long term, low dose protocol. The secondary outcome measures included potential changes in neurological and psychomotor status of PMT-treated LOTS patients as defined by the itemized description below.

The study was approved by the institutional IRB as well as by the Israeli Ministry of Health Review Board for clinical trials and was conducted in accordance with the Declaration of Helsinki. Written informed consent was obtained from each individual participating in the study.

### Neurological and psychiatric evaluation

Disability was assessed by the Standard ambulation index [12] and the Amyotrophic Lateral Sclerosis Functional Rating Scale (ALSFRS) [13]. The “up & go” test was used to measure mobility. Handgrip strength was assessed by means of dynamometry in both hands. Manual muscle strength was assessed using the modified Medical Research Council (MRC) scale. The assessed muscle functions included shoulder abduction, elbow flexion and extension, finger extension, hip flexion, knee extension and flexion and foot dorsiflexion. Muscle strength was rated for right and left side and expressed as means of the two sides. Gait and balance were assessed by the Tinetti Gait and Balance Scale [14], and the Modified Falls Efficacy Scale [15].

Psychiatric evaluation was carried out by patient interview on each visit by the same psychiatrist. Diagnosis was made according DSM IV-R criteria. The Hamilton questionnaire (HAM-21) was used for depression rating and the PANSS questionnaire was used for the evaluation of psychosis.

### HexA activity

Lymphocyte HexA enzymatic activity was determined with 4-methylumbelliferyl-6-sulfo-*N*-acetyl-ß-D-glucosaminide (MUGS) as a fluorogenic substrate as previously described [16]. In brief, cell extracts were prepared from whole blood on the day of blood collection using standard methods and stored in aliquots at −20°C. To determine HexA activity, 4 μg of lymphocyte extract protein were incubated with MUGS (0.7 mM; Melford Laboratories, Suffolk, UK) in a 50 mM citrate buffer (pH 4.5; 37°C) over 1.5 hour. Fluorescence of the liberated 4-methylumbelliferone was measured by Synergy 2 fluorometer ((BioTek Instruments, Inc Winooski, US; excitation 335 nm, emission 442 nm) and normalized for protein content [17]. To minimize inter-assay variations, each determination throughout the study also included extracts from the baseline sample and at least one additional sample from the intervention period. As variations in the ratio between this “historical” and baseline activity on repeat measurements did not exceed ±/−10%, assessment of the current (treatment) sample relative to baseline activity for each sample could be safely made within the same assay. Extracts were stable in terms of enzyme activity for at least one year. Because the study lasted for ~1.5 years, new extracts were prepared and compared with the original baseline, so as to form a credibly stable extract for continued comparative assessment. Calculated intra-assay imprecision was 10.5% and inter-assay imprecision was 26%. Biological variation in heterozygotes was 2.1%.

#### Statistical analysis

All endpoints and data were summarized using descriptive statistics (mean values with standard deviations) and non-parametric t-test using SPSS. HexA activity was calculated and presented as percent change from baseline.

## Results

### Baseline clinical assessment

Baseline demographic and clinical characteristics are presented in Table 1. Mean patient age was 28.6 (±5.3; [SD]) years and the mean duration of the pre-trial symptomatic disease was 16.4 (±3.8; [SD]) years. Mean age of first clinical sign was 12.3 (±3.2 [SD]). There was a considerable variability in the severity of the neurological impairment of the patients at the entry point to this study.

**Table 1:** **Clinical and demographic characteristics of the study group**

**Patient**	**Gender**	**Age (Years)**	**Age at first symptom (Years)**	**Symptoms at diagnosis**	**Current treatment**	**Affected family members**
1	Man	34	15	Depression, hypersomnia	Miglustat, Lithium, Sertraline, Carbamazepine Propanolol, L-thyroxine	None
2	Man	21	10	Attention deficit	None	One brother
3	Man	29	15	Psychotic episodes	Olanzapine, Lithium	One sister
4	Man	30	9	Speech problems	Olanzapine, Duloxetine	None

### Effects of pyrimethamine on HexA activity

Mean baseline HexA activity of study subjects was 72.1nmole/mg protein/hour (20.1 ± SD) (range: 48.3 to 92.1 nmol/mg protein/hour). When “normalized” in relation to activity in unaffected normal human subjects, this amounted to ~ 1.1% of the activity determined in non-carrier, healthy subjects (±0.3%; [SD]; [range: 0.7% to 1.3%); (Table 2).

**Table 2:** **HexA activity of the study group**

**Patient**	**Baseline activity nmol/hour/mg protein**	Baseline HexA activity, (% of wild type*)	Peak HexA activity (% of wild type*)	**Peak increase in HexA activity (fold Increase) relative to to baseline**
1	92.1	1.3	2.5	1.87
2	84.8	1.2	2.5	2.1
3	63.3	0.9	1.8	2
4	48.3	0.7	2.1	3

HexA activity in lymphocyte was determined using MUGS as an artificial substrate as described in the Methods. [% of wild type*] refers to mean activity in lymphocyte preparation from unaffected healthy subjects (“wild type”): 6900 ± 996 nmol/mg protein/hour (n = 3). Relative change in activity refers to fold change relative to baseline, where baseline activity or activity unchanged from baseline is referred to as 1 (no change), 2 fold describes doubling, 3 fold refers to tripling, etc.).

PMT increased peripheral lymphocyte HexA activity in all 4 study subjects with a mean peak increase of 2.24 (±0.52; [SD] folds over baseline; (range: 1.87-3). Hence, while statistically significant, this achieved peak activity equaled, on the average, to just ~ 2.2 % (±0.3%; [SD]) of the normal activity in unaffected healthy subjects.

**Figure 1:**
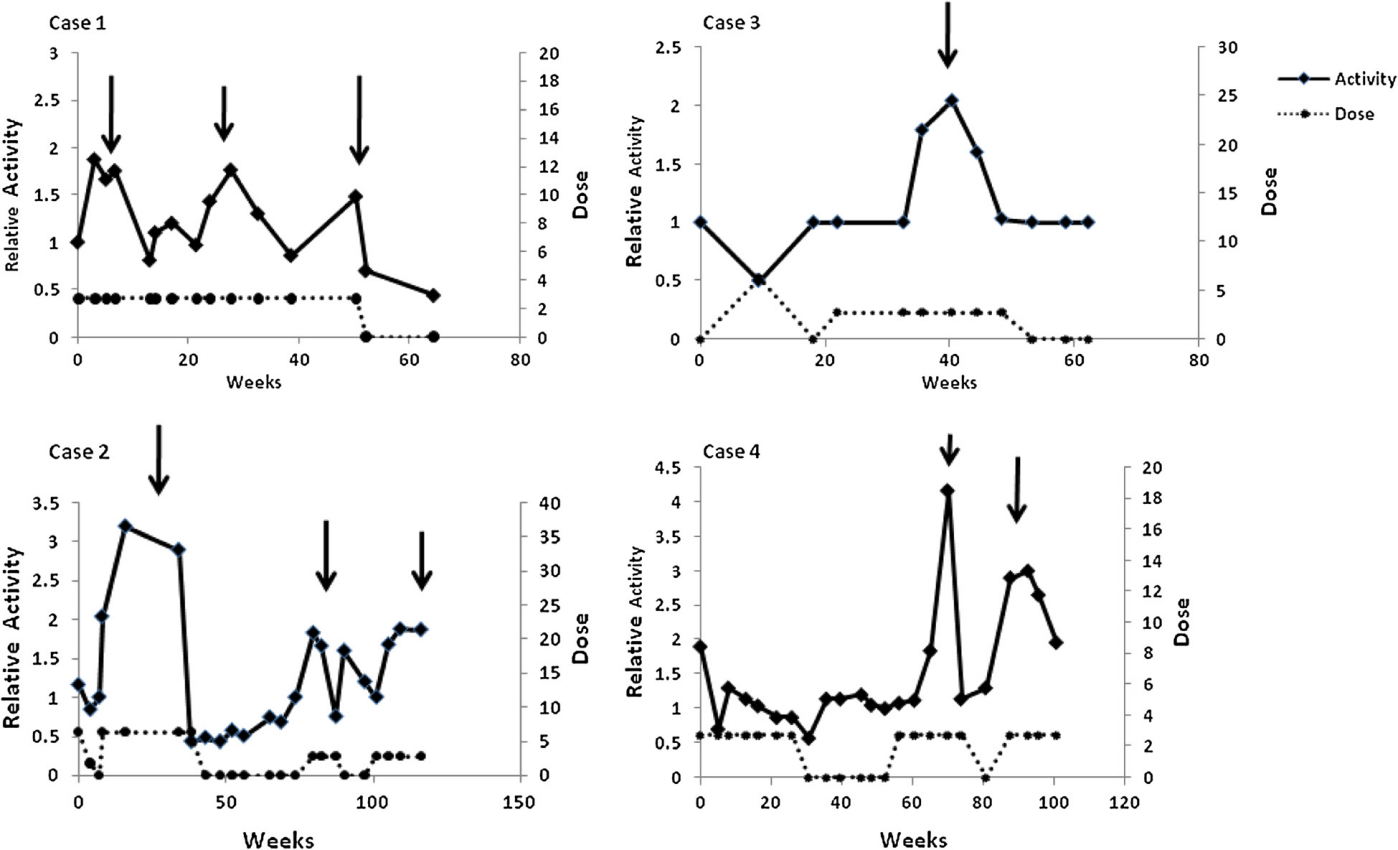
The continuous relation between HexA activity (solid line) and PMT treatment (dashed line), as a function of time. HexA activity is presented in relation to the baseline measurements, determined repeatedly with each measurement throughout the study.

Figure 1 depicts individual HexA activity throughout the study period, as a function of treatment period and dose. As can be seen, an increase in HexA activity was induced by PMT at a dose of 6.25 mg three times a week in all study subjects. The effect of PMT treatment appeared biphasic: an initial increase in HexA activity followed by a second phase of decline and inhibition of HexA activity, which was resolved by discontinuation of PMT treatment. Mean duration of a cycle was 33.4 (±15.1 SD) weeks (range: 17–52 weeks). Following drug withdrawal, once HexA activity returned to its baseline level, a second cycle of PMT was initiated and resulted again in enhancement of HexA activity followed again by a decline, quite reminiscent of the reduction in activity already observed with continuous treatment in our previous study [10]. Mean time to the first or second activity peak was 15.7 (±4.8; [SD]; range: 14.7-22 weeks). Mean time from the observed activity peak to an appreciable decrease in activity was 16.7 (±11.7 [SD]; range: 3.8-36.7) weeks. There was no differences between “time to peak” and “time to decrease” (P = 0.84), but this reflected a very large inter-patient variability, particularly in the rate at which the inhibitory effect/loss of effect evolved.

### Neuropsychiatric outcome

In general, there was large variability among the repeated assessments for all tests, which, in our opinion, was highly dependent on concomitant fluctuation in neuropsychiatric state. Still, comparison between the baseline and final visit showed no improvement in any of the study subjects. Only one subject enjoyed a crudely stable neurological state (case number 2). The other three patients experienced a variable, albeit clear neurological deterioration. In one subject (case number 3) all categories of the neurologic assessment were stable with the exception of a worsening in dysarthria, which was also the main presenting symptom of this patient. The two remaining patients, (cases 1 and 4) experienced continuous deterioration. Patient 1 had a gradual reduction in both hand grip strength and manual muscle strength, along with worsening of balance and gait ability as assessed by the Tinetti balance & gait scores. Patient 3 experienced a reduction in hand grip strength and manual muscle strength along with worsening of performance in the up & go test, the Tinetti balance & gait scores and the falls efficacy assessment.

### Adverse events

The lower doses of PMT were well-tolerated and none of the subjects experienced significant side-effects. There was no hematological or clinical evidence of folate deficiency.

## Discussion

In two previous reports (10, 11), including one from our group, it appeared that PMT could increase HexA activity in LOTS patients, thus raising some hope that this drug might be of benefit. We expressed some skepticism regarding PMT in LOTS in our first report, since we witnessed that following an initial rise in HexA, levels declined. In an attempt to avoid this secondary decline, which appeared both/either time and/or dose depended, we designed this present smaller study, using lower doses and a cyclic mode of therapy. The main finding in this study is that long term (~1.57 year) cyclic low dose PMT treatment had a biphasic, effect on HexA activity with initial enhancement followed by a second phase of inhibition of activity. However, these effects on HexA activity were somewhat variable in magnitude and duration and were not associated with discernible clinical benefits.

The increase attained by cyclic treatment PMT on HexA in LOTS patient was small to moderate, with an average rise of ~2 fold over baseline activity, reflecting a broad range of variability (1.8-3fold increase). This effect still falls short of reaching the theoretically desirable increase in HexA to “10% of normal HexA”, above which a disease-free phenotype has been documented despite the presence of LOTS mutations [2,3]. These modest results are nevertheless in general agreement with the *in vitro* study by Megawa et al. [9], which demonstrated that PMT can function as an effective pharmacological chaperone for several α- and β-mutants affecting Hex A: some, but not for all of HEXA and HEXB mutants showed a 2–3 fold increase in HexA activity in the presence of PMT. In patients carrying the G269S mutation, such as also present in all our patients, a 2 fold enhancement in HexA activity was observed. While our results in living subjects with LOTS are unfortunately disappointing, they are nevertheless useful to students in the field to prompt further the search for alternative molecules or treatment protocols.

Several aspects of the present trial outcome should be particularly noted. First, the increase in HexA activity was time dependent, although the time required to achieve the maximal response varied considerably among the study subjects. Because the same dose was used in all subjects, it would be difficult to establish whether these differences reflect variation in the HexA’s sensitivity to PMT or to differences related to the drug’s kinetics. In referring to drug kinetics, however, we note that the peak HexA activity achieved with the low dose of PMT used in the present study was not appreciably different and in fact appears somewhat higher than that achieved by us in our previous study (2.2 fold in this study; 1.78 fold in the previous study), in which much higher doses (up reaching 75 mg/day) were used [10]. This would suggest that a simple correlation between PMT levels and lymphocyte HexA activity in LOTS is highly unlikely. Second, even a very low dose of PMT such as used in the present report invariably induced, with the passage of time, a secondary decline in HexA activity. Hence, long term treatment with PMT, even at a low dose, eventually resulted in inhibition of HexA activity. Furthermore, discontinuation of PMT reversed the inhibition of HexA activity. Third, despite the observed PMT-induced clear increase in HexA activity in each of the 4 of the treated LOTS patients, and despite the fact that we were able to maintain LOTS patients on cyclic PMT treatment for up to ~1.5 year in terms of biochemical and clinical tolerability, no obvious clinical effect was observed. Three out of the four subjects in this trial experienced a continuous neurological decline. Only the youngest patient with the shortest period of disease presentation had a stable neurological state over the observed treatment period. This overall downhill course is consistent with the report of Shapiro et al. in a large trial of miglustat over a similar time interval in LOTS patients [18].

Several confounders may be related to the absence of clearly discernible neuropsychiatric benefits seen with PMT in LOTS despite some rise in peripherally measured lymphocyte HexA activity. Pre-trial neurological impairment may have been too advanced at the onset of the trial. This interpretation is supported by the finding that the single subject exhibiting disease stability throughout the trial period was also the youngest and healthiest patient in this cohort. Additionally, the induced rise in HexA may have been insufficient in terms of maximal achieved activity level and/or the duration of the attained increase. Further, whereas lymphocyte HexA activity may correlate, to some extent, with brain HexA activity, it is unlikely to accurately reflect the CNS enzyme function or dysfunction. Lymphocytes are short living cells and their exposure time to PMT is much shorter than that of the neurons positioned permanently in the brain. Indeed, in one study in human subjects receiving PMT and subjected to neurosurgical procedures, the ratio of brain parenchymal-to-serum PMT concentrations was 2.5 to 5 [6]. Additionally, PMT is a rather lipophylic drug, with prolonged half-life in humans (t1/2 = 96 hours) [6]. PMT storage in the lipophylic brain tissue may provide a continuous local source of the drug, which could eventually induce inhibition of HexA activity in neurons, in a timetable which is not necessarily reflected in peripherally measured enzyme activity. Therefore, the possibility that even a low dose PMT can partially or transiently inhibit CNS HexA can be neither excluded nor substantiated by the present means of follow up. We cannot eliminate the possibility that a small treatment effect might not have been detected, due to the obvious limitations of this study, including the small number of patients studied, the open-label design of the trial and the variability in the HexA response. The difficulty of assessing the slowly progressive clinical course in LOTS is also complicated by swings of mood which effect clinical evaluation [2]. An important additional cautionary note is that since all our subjects had the same genotypic makeup, it is still possible that PMT could do better in other genetic LOTS variants.

In the present report we assessed HexA activity using a standard assay utilizing a synthetic substrate (MUGS). While generally reliable, this widely used assay has several inherent limitations which could have affected the measurements in the present study. First, PMT concentration in the actual assay is likely to have been diluted in the course of the preparatory phases of lymphocyte isolation. Second, the assay’s pH is higher than would be expected in the lysosomes, the site of HexA activity in intact cells *in vivo*. Third, HexA activity as assessed by MUGS can reflect both HexA and HexS activity, as their abundance can be increased by PMT in Tay Sachs cells, including cells carrying the G269S nutation [9].

Finally, although pharmacological chaperones may comprise promising therapeutic tools in lysosomal diseases, the present report is not the only example of limited success in their application. Disappointing results were also seen in a phase 2 trial with the pharmacological chaperone Plicera (afegostat tartrate) in the treatment of type 1 Gaucher disease: whereas all patients enrolled in this study experienced an increase in the level of the target enzyme glucocerebrosidase as measured in white blood cells, clinically meaningful improvement was recorded in just one out of the eighteen patients completing who completed the study [19].

## Conclusion

As assessed by enzyme activity in peripheral lymphocytes, low PMT doses administered in a cyclic manner dictated by monitoring of lymphocyte HexA activity can lead to increase this enzyme’s function in LOTS patients for a period of several weeks to months. However, this trial of PMT in LOTS did not show discernible benefits of the treatment on the other measured outcomes over ~18 months treatment. These findings do not support the use of low doses of PMT for treatment of LOTS, with the potential exception of patients who are still in a very early stage of their diseases.
